# The effect of diabetes on mortality in critically ill patients: a systematic review and meta-analysis

**DOI:** 10.1186/cc10440

**Published:** 2011-09-13

**Authors:** Sarah E Siegelaar, Maartje Hickmann, Joost BL Hoekstra, Frits Holleman, J Hans DeVries

**Affiliations:** 1 Department of Internal Medicine, Academic Medical Center, Meibergdreef 9, 1105 AZ, Amsterdam, The Netherlands

**Keywords:** diabetes mellitus, critically ill, ICU mortality, in-hospital mortality, 30-day mortality, meta-analysis

## Abstract

**Introduction:**

Critically ill patients with diabetes are at increased risk for the development of complications, but the impact of diabetes on mortality is unclear. We conducted a systematic review and meta-analysis to determine the effect of diabetes on mortality in critically ill patients, making a distinction between different ICU types.

**Methods:**

We performed an electronic search of MEDLINE and Embase for studies published from May 2005 to May 2010 that reported the mortality of adult ICU patients. Two reviewers independently screened the resultant 3,220 publications for information regarding ICU, in-hospital or 30-day mortality of patients with or without diabetes. The number of deaths among patients with or without diabetes and/or mortality risk associated with diabetes was extracted. When only crude survival data were provided, odds ratios (ORs) and standard errors were calculated. Data were synthesized using inverse variance with ORs as the effect measure. A random effects model was used because of anticipated heterogeneity.

**Results:**

We included 141 studies comprising 12,489,574 patients, including 2,705,624 deaths (21.7%). Of these patients, at least 2,327,178 (18.6%) had diabetes. Overall, no association between the presence of diabetes and mortality risk was found. Analysis by ICU type revealed a significant disadvantage for patients with diabetes for all mortality definitions when admitted to the surgical ICU (ICU mortality: OR [95% confidence interval] 1.48 [1.04 to 2.11]; in-hospital mortality: 1.59 [1.28 to 1.97]; 30-day mortality: 1.62 [1.13 to 2.34]). In medical and mixed ICUs, no effect of diabetes on all outcomes was found. Sensitivity analysis showed that the disadvantage in the diabetic surgical population was attributable to cardiac surgery patients (1.77 [1.45 to 2.16], *P *< 0.00001) and not to general surgery patients (1.21 [0.96 to 1.53], *P *= 0.11).

**Conclusions:**

Our meta-analysis shows that diabetes is not associated with increased mortality risk in any ICU population except cardiac surgery patients.

## Introduction

The proportion of patients with diabetes admitted to the ICU is growing as a result of the worldwide increase in type 2 diabetes. In cardiac surgery patients, the growth is even more pronounced, since patients with diabetes-associated, often complex, multivessel macrovascular complications are preferably treated surgically rather than by percutaneous intervention [1,2]. It is known that diabetic patients admitted to the ICU are more prone to develop complications [3-5], at least in part because of hampered immune cell function associated with the disease [6,7]. One would expect an increased mortality rate for diabetic ICU patients, but the literature at this point is conflicting, with reports of increased, equal or even decreased mortality rates among this population compared to patients without diabetes. Also, there might be a difference in outcomes between various ICU populations, such as cardiac surgery and medical patients.

To better understand the role of diabetes in critical illness, we conducted a systematic review and meta-analysis of studies regarding mortality, including observational as well as intervention studies that reported ICU, in-hospital and/or 30-day mortality rates of ICU-admitted patients with diabetes.

## Materials and methods

This study was conducted according to the recommendations of the Meta-analysis of Observational Studies in Epidemiology (MOOSE) group [8]. The study did not need ethical approval, since it was a retrospective analysis of anonymous data.

### Data sources and search strategy

Together with the clinical librarian at our institution, an electronic search of MEDLINE and Embase from 1 May 2005 to 1 May 2010 was performed for studies that reported mortality of adult ICU patients, including both retrospective and prospective studies and both observational and intervention studies. The five-year limit was chosen because we expected that insulin treatment regimens and other therapies would be comparable among studies in this rapidly evolving field. The text terms and medical subheading, or MeSH, terms 'intensive care unit', 'critical care' and 'mortality' were combined. Since a preliminary search showed that diabetes was not always the primary interest of included studies, and to prevent publication bias, we narrowed the search by not using 'diabetes mellitus' as a search term. To avoid possible treatment-induced bias, randomized controlled trials comparing intensive insulin therapy regimes were excluded [9]. We limited our search to research performed in adult human patient populations and published in the English-language literature. Unpublished studies were not identified.

### Study selection

Two reviewers (SES and MH) independently screened the records. Agreement on final inclusion was reached by consensus. Inclusion and exclusion criteria were defined *a priori*. A study was included if it reported crude ICU, in-hospital and/or 30-day mortality of ICU-admitted patients with or without diabetes and/or included univariate or multivariate analysis of mortality risk for diabetic patients represented as odds ratios (ORs), hazard ratios or relative risks. Studies reporting 28- or 30-day mortality rates were combined. We excluded studies in which diabetes was the reason for ICU admittance, that is, ketoacidosis, as well as studies concerning patients with gestational diabetes and ICU readmissions. When it was not possible to obtain the full paper from our institutional library or the Internet, the corresponding author was contacted if the population included in the study was larger than 500 patients.

### Data extraction

From the included publications the following data were extracted: first author, year of publication, country where the work was performed, study design, type of ICU, population specification, reported mortality type, definition of diabetes and the numbers of patients with and without diabetes. Subsequently, the number of deaths among patients with or without diabetes and/or the mortality risk associated with the presence of diabetes were extrapolated. We contacted the corresponding author for additional information when the publication reported only a *P *value for mortality risk or when the exact number or proportion of patients with diabetes was not reported.

### Study quality

No individual assessment of study quality was performed. Funnel plot analysis did not suggest any publication bias. We did not expect bias in outcome reporting, because death is a robust end point, and it is unlikely that patients were lost to follow-up, as the primary outcome was recorded in the hospital.

### Data synthesis and statistical methods

The meta-analysis was performed using RevMan version 5 software (The Cochrane Collaboration, Oxford, UK). Analyses were performed separately for ICU, in-hospital and 30-day mortality as outcome variables. Data were synthesized using inverse variance with ORs as the effect measure. In the primary analysis, unadjusted results were used where possible. If only crude mortality data were provided, then the ORs and standard errors were calculated. An OR > 1 suggested that diabetes was associated with an increased risk of death. We stratified the analyses by ICU type: trauma, surgical, medical and mixed. Data were pooled using a random effects model because heterogeneity between studies was anticipated. Heterogeneity was assessed using the *I*^2 ^statistic.

We performed sensitivity analyses to explore the robustness of the data and the influence of the following factors on effect size by repeating the analysis: (1) using a fixed instead of a random model, (2) leaving out the two largest studies, (3) making distinctions between cardiac and other types of surgery, (4) selecting and pooling only the five studies reporting unadjusted as well as adjusted outcomes and comparing these two effect sizes to assess the possible influence of confounders and (5) using adjusted instead of unadjusted effect sizes of those five studies in the in-hospital mortality analysis.

## Results

**Figure 1:**
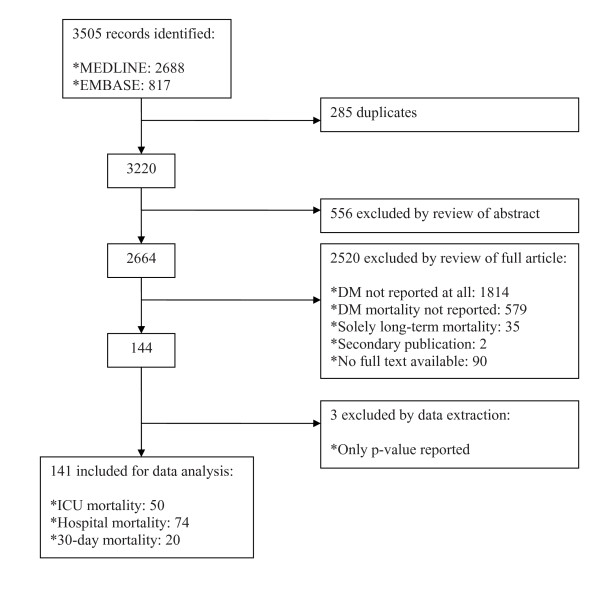
**Study selection algorithm**.

Figure 1 summarizes the study identification and selection process. After removal of duplicates derived from the Embase and MEDLINE searches, 3,220 potentially interesting records were identified. Of these, we excluded 556 publications by reviewing the abstracts. After review of 2,664 full articles, 2,520 were excluded: 1,814 because no data at all were available regarding diabetes, 579 because mortality data were not available, 35 because they reported only long-term mortality and 2 duplicate publications were identified. We were not able to obtain the full texts of 90 potentially relevant records, of which 14 reported a sample size of more than 500 participants. Three records were excluded by data extraction because only a *P *value for mortality was reported and, after contacting the authors, the raw data could not be retrieved [10-12]. Finally, 141 studies could be included in the analysis of ICU mortality (*n *= 50 [13-62]), in-hospital mortality (*n *= 74 [26,63-135]) and 30-day mortality (*n *= 20 [65,128,136-153]). Three studies reported results of two outcome types: one comprised both ICU and in-hospital mortality [26] and two comprised both in-hospital and 30-day mortality [65,128]. Only a few studies specified the type of diabetes of the patients included (*n *= 26). In most of these studies, diabetes was defined by the use of glucose-lowering drugs. A table listing the patient characteristics in the 141 included studies is presented in Additional file 1.

### ICU mortality

**Figure 2:**
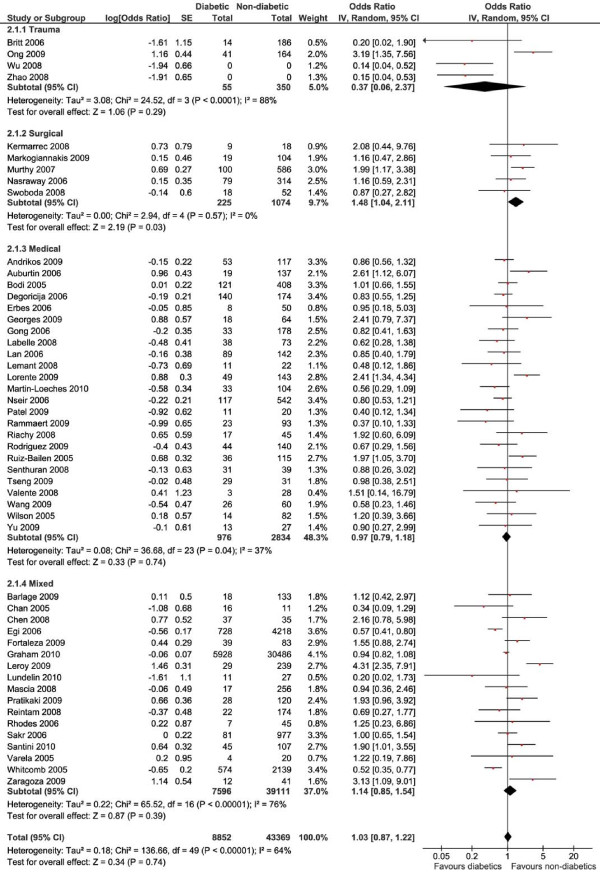
**ICU mortality**. Forest plots showing odds ratios (ORs) and 95% confidence intervals (95% CIs) of ICU mortality risk for patients with or without diabetes. The '0' indicating the number of diabetic or nondiabetic patients means the information was not available. SE: standard error. IV: inverse variance.

The analysis of ICU mortality contained 52,908 patients, including 7,576 deaths (Figure 2). Of those patients, at least 8,852 were diagnosed with diabetes (16.7%). In two studies, the number of patients with diabetes could not be retrieved [59,62]. Overall, pooling of the data showed no survival advantage for either group (OR 1.03, 95% confidence interval (95% CI) 0.87 to 1.22, *P *= 0.74, *I*^2 ^= 64%). Analysis by ICU type showed that in the surgical ICU, patients without diabetes did have a survival advantage over patients with diabetes (OR 1.48, 95% CI 1.04 to 2.11, *P *= 0.03, *I*^2 ^= 0%) No differences were observed in the trauma, medical and mixed ICUs. Overall mortality was 26.3% in general and/or cardiac surgical ICUs, 12.2% in trauma ICUs, 35.9% in medical ICUs and 11.8% in mixed ICUs.

### In-hospital mortality

**Figure 3:**
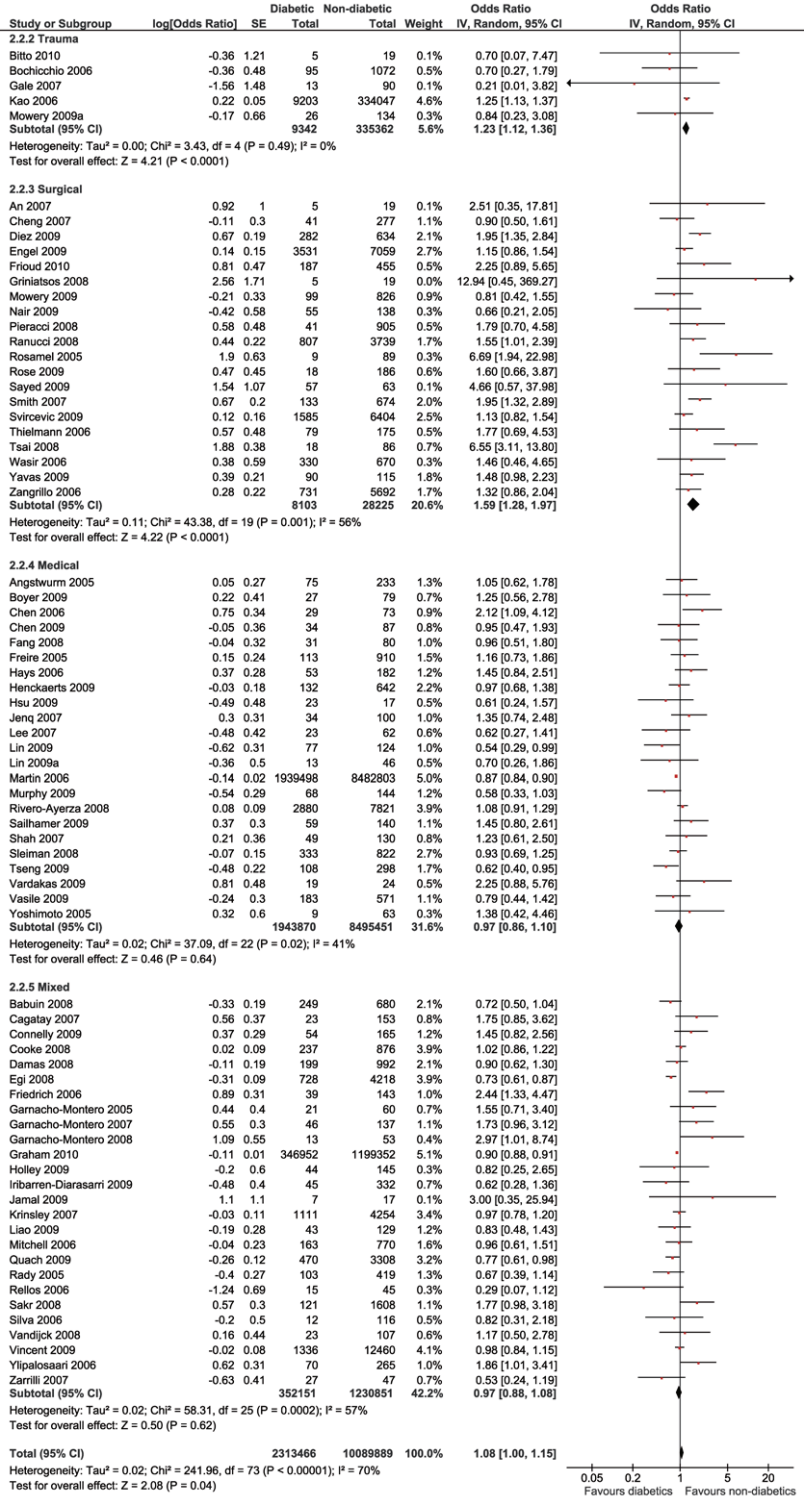
**In-hospital mortality**. Forest plots showing odds ratios (ORs) and 95% confidence intervals (95% CIs) of hospital mortality risk for patients with or without diabetes. SE: standard error. IV: inverse variance.

The largest cohort could be analysed for in-hospital mortality: 12,403,355 patients (mortality rate 21.7%), of whom 2,313,466 patients (18.7%) had diabetes (Figure 3). Pooling of all data did show a small mortality increase among the patients with diabetes (OR 1.08, 95% CI 1.00 to 1.15, *P *= 0.04, *I*^2 ^= 70%). The disadvantage for patients with diabetes was attributable to the effect in the surgical patients (OR 1.59, 95% CI 1.28 to 1.97, *P *< 0.0001, *I*^2 ^= 56%) and trauma patients (OR 1.23, 95% CI 1.12 to 1.36, *P *< 0.0001, *I*^2 ^= 0%) after stratifying by ICU type. In the medical and mixed ICUs, no difference in outcomes between the groups was seen. Overall mortality was 3.8% in surgical ICUs, 5.1% in trauma ICUs, 24.2% in medical ICUs and 9.8% in mixed ICUs.

### Thirty-day mortality

**Figure 4:**
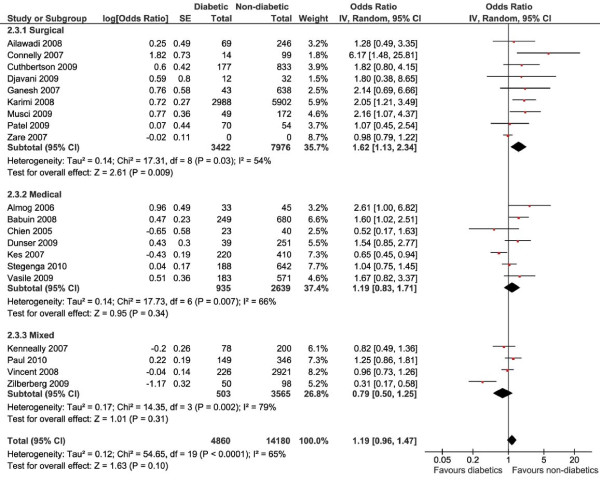
**Thirty-day mortality**. Forest plots showing odds ratios (ORs) and 95% confidence intervals (95% CIs) of 30-day mortality risk for patients with or without diabetes. The '0' indicating the number of diabetic or nondiabetic patients means the information was not available. SE: standard error. IV: inverse variance.

A total of 4,860 patients (25.5%) with diabetes and 14,180 patients without diabetes were included in this analysis (Figure 4). For one study that included 14,271 patients, the proportions of patients with or without diabetes could not be retrieved [152]. Overall, there were no differences in mortality between the patients with or without diabetes (OR 1.19, 95% CI 0.96 to 1.47, *P *= 0.10, *I*^2 ^= 65%). Stratifying the data for ICU type showed a difference in mortality favouring the patients without diabetes in the surgical ICU (OR 1.62, 95% CI 1.13 to 2.34, *P *= 0.009, *I*^2 ^= 54%). Outcomes did not differ between groups in the medical and mixed ICUs. No separate analysis of trauma patients could be performed, since none of the studies reported 30-day mortality outcomes in a trauma ICU. Overall mortality was 2.5% in surgical ICUs, 34.6% in medical ICUs and 26.9% in mixed ICUs.

### Sensitivity analyses

For ICU and 30-day mortality, the effect pointed in the same direction when using a fixed instead of a random model. No benefit was observed for patients with or without diabetes in the trauma ICUs, medical ICUs and mixed ICUs, but there was a significant disadvantage for surgical patients with diabetes. Regarding in-hospital mortality, the effect in the mixed and medical ICUs shifted towards an advantage for diabetes patients (medical: OR 0.89 [95% CI 0.85 to 0.92], *P *< 0.00001; OR mixed: 0.90 [95% CI 0.88 to 0.92], *P *< 0.00001). This effect was attributable to the weight of two very large studies. The medical ICU population in the study of Martin and colleagues [101] had an overall weight of 17.5%; The mixed ICU population in the study of Graham and colleagues [26] had an overall weight of 69.9%. Performing the fixed analysis without these two large studies resulted in the same conclusions as the random analysis, namely, no effect.

For all outcomes, we observed a mortality benefit for nondiabetic subjects in the surgical ICU. This population consisted of cardiac surgery patients as well as general surgery patients, who are quite distinct regarding the aetiology of their underlying disease compared to other ICU populations. We performed a sensitivity analysis to investigate whether the effect of diabetes might be different for cardiac surgery patients vs. general surgery patients. Since the effect was the same for ICU-, in-hospital and 30-day mortality outcomes and to increase power, we performed the sensitivity analysis by combining all studies of surgical ICU patients, regardless of mortality definition. This analysis showed that the increased mortality of patients with diabetes in the surgical ICU was mainly attributable to the 21 studies including cardiac surgery patients (OR 1.77, 95% CI 1.45 to 2.16, *P *< 0.00001, *I*^2 ^= 49%). Crude mortality was 5.1% for patients with diabetes and 3.3% for patients without diabetes. Pooling of the general surgery study data showed a trend towards higher mortality in diabetic patients (OR 1.21, 95% CI 0.96 to 1.53, *P *= 0.11, *I*^2 ^= 30%).

Most studies reported only crude mortality data or unadjusted regression analyses. To assess the possible influence of confounders, we first looked at the data derived from five studies that reported unadjusted as well as adjusted results for in-hospital mortality [26,65,75,95,121]. Among other parameters, these studies adjusted for severity of disease. Overall, no difference in effect size was observed after pooling the unadjusted results (OR 1.06 [95% CI 0.83 to 1.35]) vs. the adjusted results (OR 1.02 [95% CI 0.79 to 1.34]). Introduction of adjusted instead of unadjusted data in the analysis of in-hospital mortality did not change the outcome, except for a shift towards nonsignificance in the analysis of the trauma patients (1.02 [95% CI 0.93 to 1.13], *P *= 0.45, *I*^2 ^= 0%).

## Discussion

Our large meta-analysis shows no significant overall difference in mortality between critically ill patients with or without diabetes, except for the subgroup of cardiac surgery patients. We found a survival disadvantage for patients with diabetes admitted to the ICU after cardiac surgery. Our sensitivity analyses show good robustness of the data. If anything, using a fixed model shifts the outcome from equal survival towards a small benefit for diabetic patients in the medical and mixed ICU cohorts for in-hospital mortality, which can be attributed to the inclusion of two very large cohorts [26,101]. The clinical consequences are that patients with diabetes should not be denied access to ICUs on the basis of their diabetes. It is not possible, however, to draw conclusions regarding the possible benefits of tight glycaemia control in patients with diabetes on the basis of our meta-analysis.

It did not come as a surprise to us to find a negative impact of diabetes on the survival of cardiac surgery patients. The diabetic cardiac surgery population is different from the nondiabetic cardiac surgery population. As a result of their diabetes, diabetic cardiac surgery patients are more likely to have three-vessel coronary artery disease, left main coronary artery stenosis and left ventricular systolic dysfunction [154,155], all of which are associated with worse outcomes. Also, these patients are at increased risk for sternal wound infection, probably related to immune cell dysfunction associated with the disease [6,7].

On the other hand, it is remarkable to find no increased mortality for patients with diabetes in the medical and mixed ICU patient populations. Patients with diabetes are known to have a higher chance of developing complications such as sepsis and acute organ failure when they are admitted to the ICU [3-5]. These complications are associated with mortality, at least in the nondiabetic patient population. Apparently, diabetes is a risk factor only for the development of complications, but once acquired, the mortality risk is equal to or perhaps even lower than that of nondiabetic patients.

A possible explanation for the relative protection of critically ill patients with diabetes could lie in the different effects of stress-induced hyperglycaemia between the two groups. Hyperglycaemia is common in critically ill patients, and not only in those with diabetes. It is associated with mortality [156], but it has been shown that patients with diabetes are less affected than nondiabetic patients by high glucose levels [26,77,157,158]. It might be that the relative protection from stress-induced hyperglycaemia counteracts the increased mortality risk due to an increased amount of complications. Other mechanisms may, of course, contribute [26]. Further studies are needed to unravel the exact effects of hyperglycaemia in patients with or without diabetes.

Because of the nature of the included studies, we mainly show unadjusted results. It might be possible that the baseline characteristics between patients with or without diabetes were different, which would influence the outcome. If there were a difference, it would seem likely that patients with diabetes would be more severely ill at the time of admission. Adjusting for severity of illness would have decreased the ORs, as shown in the sensitivity analysis, thus indicating a greater advantage only for patients with diabetes. This effect is seen in the in-hospital mortality outcomes among the trauma patient population. It might be possible that adjustment for severity of disease decreases the negative effect of diabetes seen in cardiac surgery patients. However, our results represent the mortality risk of the average patient with or without diabetes, irrespective of the differences between the populations associated with diabetes.

There are limitations associated with our meta-analysis. First, the published data do not allow differentiating type 1 from type 2 diabetes or insulin-treated from non-insulin-treated diabetes, and it might be possible that there are differences in outcomes between these groups. Furthermore, incomplete capture of diet-treated or undiagnosed diabetes in the included studies is likely, as most studies relied on the use of glucose-lowering drugs. Second, we could not retrieve mortality data from three studies. It is unlikely that including these three studies would have shifted the results to a disadvantage for patients with diabetes, since the *P *values for mortality related to the presence of diabetes in these studies were not significant. Third, for 90 potentially relevant studies, the full articles could not be retrieved. These studies comprised a total of 48,263 patients, which would have contributed only 0.39% to the total population, so we expect that this had little influence on the outcomes. Fourth, the heterogeneity among studies was quite large. Fifth, the crude mortality rates in the three surgical populations were driven by the studies included: the high mortality rate in the surgical ICUs is heavily weighted by general surgery studies, whilst for in-hospital and 30-day mortality figures, the cardiac surgery studies with much lower mortality rates dominated.

## Conclusions

We show in this meta-analysis that patients with diabetes who are admitted to medical, mixed and trauma ICUs have chances of survival similar to those of patients without diabetes. Diabetes significantly increases mortality risk only in patients admitted after surgery, more specifically after cardiac surgery, a population with distinct characteristics of the underlying disease. Further studies are needed to unravel the pathophysiological mechanisms by which patients with diabetes seem to be relatively protected in nonsurgical settings, despite higher complication rates.

## Key messages

• Patients with diabetes admitted to medical, mixed and trauma ICUs have chances of survival similar to those of patients without diabetes.

• Patients with diabetes should not be denied access to ICU facilities on the basis of their diabetes.

• The presence of diabetes significantly increases mortality risk for patients admitted after cardiac surgery.

• The pathophysiological mechanisms by which patients with diabetes have equal mortality rates despite higher complication risks in nonsurgical settings should be a subject of further research.

## Abbreviations

CI: confidence interval; DM: diabetes mellitus; OR: odds ratio; SE: standard error.

## Competing interests

The authors declare that they have no competing interests. No funding was involved in the preparation of the manuscript.

## Authors' contributions

SES contributed to the conception and design of the study; performed acquisition, analysis and interpretation of the data; and drafted the manuscript. MH contributed to the conception and design of the study, performed acquisition and analysis of the data and helped draft the manuscript. JBH contributed to the interpretation of the data and revised the manuscript for important intellectual content. FH and JHD contributed to the conception and design of the study and the interpretation of the data and revised the manuscript for important intellectual content. All authors gave their approval of the final version of the manuscript.

## Supplementary Material

Additional file 1 **Characteristics of the 141 studies included in the meta-analysis of ICU-, hospital- and 30-day mortality of patients with diabetes admitted to the ICU**. Click here for file
