# Supplementary material for: The effect of diabetes on mortality in critically ill patients: a systematic review and meta-analysis
Source: Crit Care. 2011 Sep 13;15(5):R205. doi: 10.1186/cc10440 (PMC3334749; doi:10.1186/cc10440)
Supplement: Additional file 1 — Characteristics of the 141 studies included in the meta-analysis of ICU-, hospital- and 30-day mortality of patients with diabetes admitted to the ICU. [file cc10440-S1.DOC]

Table 1; Characteristics of the studies included in the meta-analysis

| **Author** | **Year** | **Country** | **ICU type** | **Population** | **Design** | **Mortality** | **N patients** | **% DM** | **Diabetes type** |
| --- | --- | --- | --- | --- | --- | --- | --- | --- | --- |
| Ailawadi | 2008 | USA | Surgical | Mitral valve surgery + >70y | Retrospective | 30d | 315 | 21.9 | n.s. |
| Almog | 2006 | Israel | Medical | APACHE >12 | Prospective | 30d | 78 | 42.3 | n.s. |
| An | 2007 | China | Surgical | Cardiac surgery | Retrospective | Hospital | 24 | 20.8 | n.s. |
| Andrikos | 2009 | Greece | Mixed | AKI | Prospective | ICU | 170 | 31.2 | n.s. |
| Angstwurm | 2005 | Germany | Medical | Sepsis | Retrospective | Hospital | 308 | 24.4 | n.s. |
| Auburtin | 2006 | France | Medical | Pneumococcal meningitis | Prospective | ICU | 156 | 12.2 | n.s. |
| Babuin | 2008 | USA | Mixed | n.s. | Retrospective | Hospital, 30d | 929 | 26.8 | n.s. |
| Barlage | 2009 | Germany | Mixed | Sepsis | Prospective | ICU | 151 | 11.9 | IDDM + NIDDM |
| Bitto | 2010 | Italy | Trauma | Chest trauma | Prospective | Hospital | 24 | 20.8 | n.s. |
| Bochicchio | 2006 | USA | Trauma | n.s. | Prospective | Hospital | 1,167 | 8.1 | IDDM + NIDDM |
| Bodi | 2005 | Spain | Medical | CAP | Prospective | ICU | 529 | 22.9 | n.s. |
| Boyer | 2009 | France | Medical | Necrotizing soft tissue infection | Retrospective | Hospital | 106 | 25.5 | n.s. |
| Britt | 2006 | USA | Trauma | Burn | Retrospective | ICU | 200 | 7.0 | n.s. |
| Cagatay | 2007 | Turkey | Mixed | Nocosomial bacteraemia | Prospective | Hospital | 176 | 13.1 | n.s. |
| Chan | 2005 | Singapore | Mixed | Melioidosis | Retrospective | ICU | 27 | 59.3 | n.s. |
| Chen | 2006 | Taiwan | Medical | Cirrhosis | Prospective | Hospital | 102 | 28.4 | n.s. |
| Chen | 2008 | Taiwan | Mixed | Pyogenic liver abscess | Retrospective | ICU | 72 | 51.4 | n.s. |
| Chen | 2009 | Taiwan | Medical | Sepsis | Prospective | Hospital | 121 | 28.1 | n.s. |
| Cheng | 2007 | China | Surgical | Sepsis | Prospective | Hospital | 318 | 12.9 | n.s. |
| Chien | 2005 | Taiwan | Medical | Sepsis | Prospective | 30d | 63 | 36.5 | n.s. |
| Connelly | 2007 | Australia | Surgical | Aortic/mitral valve surgery | Retrospective | 30d | 113 | 12.4 | Type 1 + 2 |
| Connelly | 2009 | USA | Mixed | HAP | Retrospective | Hospital | 219 | 24.7 | n.s. |
| Cooke | 2008 | USA | Mixed | ALI | Retrospective | Hospital | 1,113 | 21.3 | n.s. |
| Cuthbertson | 2009 | UK | Surgical | Cardiac surgery | Prospective | 30d | 1,010 | 17.5 | n.s. |
| Damas | 2008 | Belgium | Mixed | Infection + OF | Prospective | Hospital | 1,191 | 16.7 | n.s. |
| Degoricija | 2006 | Croatia | Medical | Sepsis | Pro-/retrospective | ICU | 314 | 44.6 | n.s. |
| Diez | 2009 | Germany | Surgical | Valve ± CABG + renal failure | Retrospective | Hospital | 916 | 30.8 | n.s. |
| Djavani | 2009 | Sweden | Surgical | Ruptured AAA | Prospective | 30d | 44 | 27.3 | n.s. |
| Dunser | 2009 | Worldwide | Medical | Septic shock | Retrospective | 30d | 290 | 13.4 | n.s. |
| Egi | 2006 | Australia | Mixed | n.s. | Retrospective | ICU | 4,946 | 14.7 | n.s. |
| Egi | 2008 | Australia | Mixed | n.s. | Retrospective | Hospital | 4,946 | 14.7 | IDDM + NIDDM |
| Engel | 2009 | USA | Surgical | CABG | Retrospective | Hospital | 10,590 | 33.3 | n.s. |
| Erbes | 2006 | Germany | Medical | Active pulmonary TBC | Retrospective | ICU | 58 | 13.8 | n.s. |
| Fang | 2008 | Taiwan | Medical | Cirrhosis + ARF | Prospective | Hospital | 111 | 27.9 | n.s. |
| Fortaleza | 2009 | Brazil | Mixed | n.s. | Retrospective | ICU | 122 | 32.0 | n.s. |
| Freire | 2005 | USA | Medical | n.s. | Prospective | Hospital | 1,023 | 11.0 | n.s.; DKA excluded |
| Friedrich | 2006 | Canada | Mixed | n.s. | Prospective | Hospital | 182 | 21.4 | History or discharged with meds |
| Frioud | 2010 | Switzerland | Surgical | Cardiac surgery | Retrospective | Hospital | 642 | 29.1 | History or APG >11.1 mmol/l |
| Gale | 2007 | USA | Trauma | n.s. | Retrospective | Hospital | 103 | 12.6 | n.s. |
| Ganesh | 2007 | UK | Surgical | Lung transplantation | Retrospective | 30d | 681 | 6.3 | n.s. |
| Gamacho-M | 2005 | Spain | Mixed | VAP | Prospective | Hospital | 81 | 25.9 | n.s. |
| Gamacho-M | 2007 | Spain | Mixed | VAP | Retrospective | Hospital | 183 | 25.1 | Requiring treatment |
| Gamacho-M | 2008 | Spain | Mixed | Catheter related BSI | Prospective | Hospital | 66 | 19.7 | n.s. |
| Georges | 2009 | France | Medical | Acute bacterial meningitis | Retrospective | ICU | 82 | 22.0 | n.s. |
| Gong | 2006 | USA | Medical | ARDS | Prospective | ICU | 211 | 15.6 | n.s. |
| Graham | 2010 | USA | Mixed | n.s. | Retrospective | Hospital | 1,509,890 | 22.6 | ICD-9; no DKA, HONC |
|  |  |  |  |  | Prospective | ICU, hospital | 36,414 | 16.3 | n.s.; no DKA, HONC |
| Griniastos | 2008 | Greece | Surgical | Cholecystectomy | Prospective | Hospital | 24 | 20.8 | n.s. |
| Hays | 2006 | USA | Medical | Ischemic stroke | Retrospective | Hospital | 235 | 22.6 | n.s. |
| Henckaerts | 2009 | Belgium | Medical | n.s. | Retrospective | Hospital | 774 | 17.1 | Insulin, OAD or diet |
| Holley | 2009 | Worldwide | Mixed | Candidemia | Retrospective | Hospital | 189 | 23.3 | n.s. |
| Hsu | 2009 | Taiwan | Medical | Septic meningitis | Retrospective | Hospital | 40 | 57.5 | n.s. |
| Iribarren-D | 2009 | Spain | Mixed | Mixed | Prospective | Hospital | 377 | 11.9 | n.s. |
| Jamal | 2009 | Kuwait | Mixed | A. baumanii infection | Prospective | Hospital | 24 | 29.2 | n.s. |
| Jenq | 2007 | Taiwan | Medical | Cirrhosis | Prospective | Hospital | 134 | 25.4 | n.s. |
| Kao | 2006 | USA | Trauma | n.s. | Retrospective | Hospital | 343,250 | 2.7 | IDDM + NIDDM |
| Karimi | 2008 | Iran | Surgical | CABG | Cross-sectional | 30d | 8,890 | 33.6 | n.s. |
| Kemarrec | 2008 | France | Surgical | Abdominal sepsis | Prospective | ICU | 27 | 33.3 | n.s. |
| Kenneally | 2007 | USA | Mixed | C. difficile infection | Retrospective | 30d | 278 | 28.1 | n.s. |
| Kes | 2007 | Croatia | Medical | Ischemic stroke | Retrospective | 30d | 630 | 34.9 | n.s. |
| Krinsley | 2007 | USA | Mixed | n.s. | Retrospective | Hospital | 5,365 | 20.7 | n.s. |
| Labelle | 2008 | USA | Medical | Candida BSI | Retrospective | ICU | 111 | 34.2 | n.s. |
| Lan | 2006 | Taiwan | Medical | Ischemic stroke | Retrospective | ICU | 233 | 32.2 | n.s. |
| Lee | 2007 | Korea | Medical | CAP | Retrospective | Hospital | 85 | 27.1 | n.s. |
| Lemant | 2008 | France | Medical | Chikunguya infection | Prospective | ICU | 33 | 33.3 | n.s. |
| Leroy | 2009 | France | Mixed | Invasive Candida infection | Prospective | ICU | 268 | 10.8 | Type 1 |
| Liao | 2009 | Taiwan | Mixed | ARDS | Prospective | Hospital | 172 | 25.0 | n.s. |
| Lin (a) | 2009 | Taiwan | Medical | n.s. | Prospective | Hospital | 201 | 38.3 | n.s. |
| Lin (b) | 2009 | Taiwan | Medical | Pulmonary TBC + RF | Retrospective | Hospital | 59 | 22.0 | n.s. |
| Lorente | 2009 | Spain | Medical | Sepsis | Prospective | ICU | 192 | 25.5 | n.s. |
| Lundelin | 2010 | Spain | Mixed | n.s. | Prospective | ICU | 38 | 28.9 | Insulin, OAD or diet |
| Markogiannakis | 2009 | Greece | Surgical | Infection | Prospective | ICU | 123 | 15.4 | n.s. |
| Martin | 2006 | USA | Medical | Sepsis | Retrospective | Hospital | 10,422,301 | 18.6 | n.s. |
| Martin-L | 2010 | Europe | Medical | CAP + intubation | Prospective | ICU | 137 | 24.1 | n.s. |
| Mascia | 2008 | Europe | Mixed | Neurological | Retrospective | ICU | 273 | 6.2 | n.s. |
| Mitchell | 2006 | Australia/NZL | Mixed | n.s. | Prospective | Hospital | 933 | 17.5 | Incl. insulin treatment |
| Mowery (a) | 2009 | USA | Surgical | General surgery | Prospective | Hospital | 925 | 10.7 | Type 1 + 2 |
| Mowery (b) | 2009 | USA | Trauma | Brain injury | Prospective | Hospital | 106 | 16.3 | IDDM + NIDDM |
| Murphy | 2009 | USA | Medical | ALI + septic shock | Retrospective | Hospital | 212 | 32.1 | n.s. |
| Murthy | 2007 | USA | Surgical | Cardiac surgery | Retrospective | ICU | 686 | 14.6 | Insulin, OAD or diet |
| Musci | 2009 | Germany | Surgical | Endocarditic surgery | Retrospective | 30d | 221 | 22.2 | n.s. |
| Nair | 2009 | USA | Surgical | Liver transplantation | Prospective | Hospital | 193 | 28.5 | History, insulin/OAD, HbA1c >7% |
| Nasraway | 2006 | USA | Surgical | >4days ICU | Retrospective | ICU | 393 | 20.1 | n.s. |
| Nseir | 2006 | France | Medical | COPD + >48h MV | Prospective | ICU | 659 | 17.8 | n.s. |
| Ong | 2009 | USA | Trauma | >30days ICU | Retrospective | ICU | 205 | 20.0 | n.s. |
| Patel (a) | 2009 | USA | Medical | Candida septic shock | Retrospective | ICU | 31 | 35.5 | n.s. |
| Patel (b) | 2009 | USA | Surgical | Cardiac surgery + wound inf. | Retrospective | 30d | 124 | 56.5 | Insulin or OAD |
| Paul | 2010 | Israel | Mixed | Infection | Prospective | 30d | 495 | 30.1 | n.s. |
| Pieracci | 2008 | USA | Surgical | Surgery | Prospective | Hospital | 946 | 4.3 | n.s. |
| Pratikaki | 2009 | Greece | Mixed | Bloodstream infection | Prospective | ICU | 148 | 18.9 | n.s. |
| Quach | 2009 | Canada | Mixed | n.s. | Retrospective | Hospital | 3,778 | 12.4 | ICD-10 code |
| Rady | 2005 | USA | Mixed | n.s. | Retrospective | Hospital | 522 | 19.7 | n.s. |
| Rammaert | 2009 | France | Medical | COPD + MV | Prospective | ICU | 116 | 19.8 | n.s. |
| Ranucci | 2008 | Italy | Surgical | Cardiac surgery | Retrospective | Hospital | 4,546 | 17.8 | Insulin or OAD |
| Reintam | 2008 | Estonia | Mixed | n.s. | Prospective | ICU | 196 | 11.2 | n.s. |
| Rellos | 2006 | Greece | Mixed | >90y | Prospective | Hospital | 60 | 25.0 | n.s. |
| Rhodes | 2006 | UK | Mixed | n.s. | Prospective | ICU | 52 | 13.5 | n.s. |
| Riachy | 2008 | Lebanon | Medical | Severe stroke | Pro-/retrospective | ICU | 62 | 27.4 | n.s. |
| Rivero-A | 2008 | Europe | Medical | Atrial fibrillation | Retrospective | Hospital | 10,701 | 26.9 | n.s. |
| Rodriguez | 2009 | Spain | Medical | Bacterial CAP | Retrospective | ICU | 184 | 23.9 | Type 2 |
| Rosamel | 2005 | France | Surgical | Endocarditic surgery | Retrospective | Hospital | 98 | 9.2 | n.s. |
| Rose | 2009 | UK | Surgical | Massive transfusion | Retrospective | Hospital | 204 | 8.8 | n.s. |
| Ruiz-B | 2005 | Spain | Medical | AMI post thrombolysis | Retrospective | ICU | 151 | 23.8 | n.s. |
| Sailhamer | 2009 | USA | Medical | C. difficile colitis | Retrospective | Hospital | 199 | 29.6 | n.s. |
| Sakr | 2006 | Europe | Mixed | n.s. | Prospective | ICU | 1,058 | 7.7 | n.s. |
| Sakr | 2008 | Europe | Mixed | n.s. | Prospective | Hospital | 1,729 | 7.0 | n.s. |
| Santini | 2010 | Croatia | Mixed | Sepsis | Retrospective | ICU | 152 | 29.6 | n.s. |
| Sayed | 2009 | Egypt/Germany | Surgical | Cardiac surgery | Retrospective | Hospital | 120 | 47.5 | RPG ≥8.3, FPG≥7 mmol/l or meds |
| Senthuran | 2008 | Australia | Medical | ESRF | Retrospective | ICU | 70 | 44.3 | n.s. |
| Shah | 2007 | USA | Medical | n.s. | Prospective | Hospital | 179 | 27.4 | n.s. |
| Silva | 2006 | Brazil | Mixed | ARF | Retrospective | Hospital | 128 | 9.4 | n.s. |
| Sleiman | 2008 | Italy | Medical | n.s. | Retrospective | Hospital | 1155 | 28.8 | History or medication |
| Smith | 2007 | USA | Surgical | Trauma/surgery | Prospective | Hospital | 807 | 16.5 | IDDM + NIDDM |
| Stegenga | 2010 | Worldwide | Medical | Sepsis | Retrospective | 30-day | 830 | 22.7 | n.s. |
| Svircevic | 2009 | Netherlands | Surgical | Cardiac surgery | Retrospective | Hospital | 7,989 | 19.8 | n.s. |
| Swoboda | 2008 | Germany | Surgical | Sepsis/septic shock | Retrospective | ICU | 70 | 25.7 | n.s. |
| Thielmann | 2006 | Germany | Surgical | Cardiac surgery | Prospective | Hospital | 254 | 31.1 | n.s. |
| Tsai | 2008 | Taiwan | Surgical | ECMO + acute dialysis | Prospective | Hospital | 104 | 17.3 | n.s. |
| Tseng (a) | 2009 | Taiwan | Medical | Pancreatitis + RF | Retrospective | ICU | 60 | 48.3 | n.s. |
| Tseng (b) | 2009 | USA | Medical | Pneumonia | Retrospective | Hospital | 406 | 26.6 | n.s. |
| Valente | 2008 | Italy | Medical | STEMI | Prospective | ICU | 31 | 9.7 | n.s. |
| Vandijck | 2008 | Belgium | Mixed | BSI | Retrospective | Hospital | 152 | 17.7 | n.s. |
| Varela | 2005 | Spain | Mixed | MOF | Prospective | ICU | 24 | 16.7 | n.s. |
| Vasile | 2009 | USA | Medical | GI bleeding | Retrospective | Hospital, 30d | 754 | 24.3 | n.s. |
| Vincent | 2008 | Europe | Mixed | Blood transfusion | Prospective | 30d | 3,147 | 7.2 | IDDM |
| Vincent | 2009 | Worldwide | Mixed | Infection | Prospective | Hospital | 13,796 | 9.7 | IDDM |
| Wang | 2009 | Taiwan | Medical | Sepsis/septic shock | Prospective | ICU | 86 | 30.2 | n.s. |
| Wasir | 2006 | India | Surgical | Cardiac surgery | Prospective | Hospital | 1,000 | 33.0 | n.s. |
| Whitcomb | 2005 | USA | Mixed | n.s. | Retrospective | ICU | 2,713 | 21.2 | n.s. |
| Wilson | 2005 | Australia | Medical | CAP | Retrospective | ICU | 96 | 14.6 | n.s. |
| Wu | 2008 | China | Trauma | ARDS | Retrospective | ICU | 440 | unk | n.s. |
| Yavas | 2009 | Turkey | Surgical | Cardiac surgery + tracheostomy | Retrospective | Hospital | 205 | 43.9 | n.s. |
| Ylipalosaari | 2006 | Finland | Mixed | Infection + LOS >48h | Prospective | Hospital | 335 | 20.9 | n.s. |
| Yoshimoto | 2005 | Japan | Medical | CAP | Retrospective | Hospital | 72 | 12.5 | n.s. |
| Yu | 2009 | China | Medical | Septic shock | Prospective | ICU | 40 | 32.5 | n.s. |
| Zangrillo | 2006 | Italy | Surgical | Cardiac surgery | Prospective | Hospital | 6,423 | 11.4 | Insulin or OAD |
| Zaragoza | 2009 | Spain | Mixed | Candida infection | Prospective | ICU | 53 | 22.6 | n.s. |
| Zare | 2007 | USA | Surgical | Non-emergent major surgery | Retrospective | 30d | 14,271 | unk | Insulin or OAD |
| Zarrilli | 2007 | Italy | Mixed | A. baumanii infection | Prospective | Hospital | 74 | 36.5 | n.a. |
| Zhao | 2008 | China | Trauma | ARDS | Retrospective | ICU | 247 | unk | n.s. |
| Zilberberg | 2009 | USA | Mixed | C. difficile infection | Retrospective | 30d | 148 | 33.8 | n.s. |

AAA, acute abdominal aneurysm; AKI, acute kidney injury; ALI, acute lung injury; AMI, acute myocardial infarction; APACHE, acute physiology and chronic health evaluation (score); APG, admission plasma glucose; ARDS, acute respiratory distress syndrome; ARF, acute renal failure; BSI, bloodstream infection; CABG, coronary artery bypass grafting; CAP, community acquired pneumonia; DKA, diabetic ketoacidosis; DM, diabetes mellitus; ECMO, extra corporal membrane oxygenation; ESRF, end stage renal failure; FPG, fasting plasma glucose; GI, gastro-intestinal; HAP, hospital acquired pneumonia; HONC, hyperglycaemic hyperosmolar non-ketotic coma; IDDM, insulin dependent diabetes mellitus; ICD-9/10, international classification of diseases, 9^th^ or 10^th^ revision; ICU, intensive care unit; LOS, length of stay; MV, mechanical ventilation; (M)OF, (multi) organ failure; NIDDM, non insulin dependent diabetes mellitus; n.s., not specified; NZL, New Zealand; OAD, oral antidiabetic drugs; RPG, random plasma glucose; RF, respiratory failure; STEMI, ST-segment elevated myocardial infarction; TBC, tuberculosis; UK, United Kingdom; unk, unknown; USA, United States of America; VAP, ventilator associated pneumonia
